# Improving the Working Models for Drug–Drug Interactions: Impact on Preclinical and Clinical Drug Development

**DOI:** 10.3390/pharmaceutics17020159

**Published:** 2025-01-24

**Authors:** James Nguyen, David Joseph, Xin Chen, Beshoy Armanios, Ashish Sharma, Peter Stopfer, Fenglei Huang

**Affiliations:** 1 Boehringer Ingelheim Pharmaceuticals, Inc., Ridgefield, CT 06877, USA; james.nguyen2@wsu.edu (J.N.); david.joseph@boehringer-ingelheim.com (D.J.); cx2436cx@uw.edu (X.C.); beshoy.armanios@uconn.edu (B.A.); ash.sharma@boehringer-ingelheim.com (A.S.); 2 College of Pharmaceutical Sciences, Washington State University, Spokane, WA 99202, USA; 3 School of Pharmacy, University of Washington, Seattle, WA 98195, USA; 4 College of Pharmacy, University of Connecticut, Storrs, CT 06269, USA; 5 Boehringer Ingelheim Pharma GmbH & Co. KG, 88400 Biberach an der Riß, Germany; peter.stopfer@boehringer-ingelheim.com

**Keywords:** IVIVE, drug–drug interaction, excretion

## Abstract

**Background**: Pharmacokinetic drug–drug interactions (DDIs) can be caused by the effect of a pharmaceutical compound on the activity of one or more subtypes of the Cytochrome P450 (CYP) family, UDP-glucuronosyltransferases (UGTs), and/or transporters. As the number of therapeutic areas with polypharmacy has increased, interest has grown in assessing the risk of DDIs during the early phases of drug development. Various lines of research have led to improved mathematical models to predict DDIs, culminating in the Food and Drug Administration’s (FDA) guidelines on evaluating pharmacokinetic DDI risks. However, the recommended static models are highly conservative and often result in false positive predictions. The current research aims to improve the workflow for assessing CYP-mediated DDI risk using Boehringer Ingelheim (BI) proprietary compounds. **Methods**: The Drug–drug Interaction Risk Calculator (PharmaPendium) was used to evaluate the mechanistic static model, and predictions were correlated with human pharmacokinetic studies from Phase I clinical trials. **Results**: The results demonstrated that the FDA formula performed well in predicting DDIs for BI proprietary compounds. Furthermore, the integration of either human renal excretion or preclinical species total excretion data into the mechanistic static model enhanced the predictive performance for candidate drugs as victims in DDIs. **Conclusions**: The basic static models (BSMs) for drug interactions should be used in early drug discovery to “rule out” DDI risks because of the minimal inputs required and the low rate of false negative predictions. Mechanistic static models (MSMs) can then be implemented for compounds that require additional evaluation.

## 1. Introduction

Over the past decades, there has been increased interest in assessing pharmacokinetic drug–drug interaction (DDI) risks of molecules in the early phases of drug development. This is particularly important in therapeutic areas where patients are on polypharmacy, as it is imperative that potential drug candidates have minimal DDI risks which could lead to decreased efficacy or increased safety events or off-target effects of the developmental drug and/or comedications.

In drug discovery, it is recommended to promptly assess the probability of pharmacokinetic DDIs to limit the need for clinical DDI studies and to obtain a drug that patients can take without significant DDI risks. The best drug candidates typically have high pharmacological activity and low potential for DDIs. Furthermore, drug interaction risks can halt a promising compound from further clinical development. The ultimate goal in this field of research is to limit the prediction of false positive and false negative interactions, while also minimizing the resources needed to make robust DDI assessments.

Pharmacokinetic DDIs are generally caused by the effect of a drug on the activity of one or more subtypes of the Cytochrome P450 (CYP) family. This can be through either inhibition or induction of CYPs, and is most often related to the activity of CYP3A4. Besides CYPs, DDIs can be propagated through other mechanisms including attenuation of UDP-glucuronosyltransferases and drug transporters interactions [1].

In 2006, the US Food and Drug Administration (FDA) provided guidance on studying DDIs in in vitro models; the formulas for DDI predictions were similar to basic models [2]. In the following 15 years, many studies were published comparing calculated theoretical data of groups of compounds with established clinical data using various calculation methods or available software [1,3,4]. These methods to improve the in vitro to in vivo extrapolation (IVIVE) were mostly based on commercially available approved therapeutics; the FDA subsequently updated the DDI guidance (in vitro and clinical) in 2012 as the draft guideline and in 2020 as final guidelines based on these research findings [5,6]. Efforts have been made to evaluate these models of potential pharmacokinetic DDIs mediated by CYPs and/or drug transporters to improve the accuracy of IVIVEs. Shardlow et al. (2011) [3] retrospectively studied 26 small molecule drugs and drug candidates from GlaxoSmithKline with diverse physiochemical properties, including both reversible and potentially irreversible CYP inhibitors. Besides basic static models, mechanistic static models (MSMs) that differentiate between reversible and metabolism-dependent inhibition were also used, and the contribution of intestinal metabolism for CYP3A4 substrates was studied as well. The results suggested that the estimated unbound liver concentration or unbound hepatic inlet concentration, with consideration of the intestinal contribution, offered the most accurate predictions of DDIs (occurrence and magnitude) for the drugs in this dataset.

Subsequently, Peters et al. (2012) [4] used 11 AstraZeneca proprietary compounds to evaluate the effectiveness of a mathematically revised static model equation (including the hepatic first pass metabolism, etc.), and to test the applicability of SimCYP for DDIs-associated variabilities. Interestingly, the static calculations using an unbound average steady-state systemic inhibitor concentration (I_sys_) and a fixed fraction of gut extraction performed better than SimCYP (84% compared with 58% of the interactions predicted within a 2-fold range). Differences in the prediction outcomes between the static and dynamic models are attributable to differences in the first-pass contribution to DDI. These investigations support the use of static models when elimination routes of the victim compound and the role of gut extraction for the victim and/or inhibitor in humans are unknown.

In a similar approach, Einolf et al. (2014) [7] tested various DDI models for their performance in predicting DDI potential via CYP3A substrates with data from 28 clinical trials based mostly on approved drugs. All models worked with high fidelity and low false negative or false positive rates. The correlation approaches (ratio of the in vivo peak plasma concentration (C_max_) to the in vitro half-maximal effective concentration (EC_50_); and relative induction score) and basic static model (calculated R_3_-value) resulted in no false negatives when the total C_max_ was incorporated. Mechanistic static models (net effect) and mechanistic dynamic models (physiologically based pharmacokinetic modeling, PBPK) that included both CYP inactivation and induction resulted in less accurate DDI predictions, likely due to an overprediction of the effect of inactivation.

As continuous efforts are made to minimize false positive and false negative predictions, and, thus, improve overall prediction rates, research groups have been trying to improve the static models through various modifications (e.g., using higher cut-off values, refining the effective concentration of the perpetrator, etc.). For SLC transporters, Rodrigues (2023) [1] demonstrated that only compounds with high R values (>1.5) or higher maximal concentration/mean half-maximal inhibitory concentration (C_max,u_/IC_50_) ratios (>0.5) are likely to significantly influence hepatic (area under the curve ratio, AUCR > 1.25) and renal (ΔCL_renal_ > 25%) endogenous biomarkers in DDIs. Consequently, he suggested raising the cutoff values for DDI assessment to reduce the rate of false positive predictions.

Efforts in improving pharmacokinetic DDI predictions have also been made using dynamic models, where the time-course for the inhibition process as well as active metabolites are characterized and integrated into the model to generate more physiologically relevant scenarios. In a study to identify DDIs with methadone and buprenorphine, Miano et al. (2022) [8] included the factor (1 − f_e_) (f_e_ is the fraction of the target drug that is excreted unchanged in the urine) in the formula to improve the prediction. However, these modifications did not always significantly improve the predicted outcome when compared against clinical data [9]. All of these efforts to improve model predictions appear to be limited for screening of DDI potential as described in the guidelines published by the FDA in 2020 [5]. Hence, a further refinement of the models is necessary to increase the rate of true DDI predictions.

In this work, we evaluated the performance of the FDA static models (both basic model (BSM) and mechanistic static models (MSMs)) in predicting pharmacokinetic DDI risks using Boehringer Ingelheim (BI) proprietary development compounds. In addition, based on our work, we suggested an improvement in the workflow for assessing DDIs in preclinical and clinical development. The results from this study demonstrated that the FDA BSM and MSM performed well in pharmaceutical industry settings to support drug development; this supports the use of basic static models to rule-out DDI risks in the early phases of drug development, as the rate of false negative predictions are negligible. The use of the more resource-intensive mechanistic models should be reserved when additional assessments are necessary. In addition, when available, clinical renal excretion data can be incorporated into MSMs to improve the precision in predicting the compound of interest as a victim of DDIs; excretion data from preclinical species can potentially be used in lieu of clinical data to refine prediction accuracy.

## 2. Materials and Methods

For the current investigation, a total of 44 clinical studies were used to assess the predictive performance of basic and MSMs outlined in the FDA guidance on in vitro drug interaction studies [5]. These studies encompass 18 BI proprietary compounds that have clinical DDI data. The dataset includes small molecules with a diverse range of physicochemical properties, spanning various therapeutic areas. BI compounds were assessed as both the object drug and precipitant of reversible inhibition, time-dependent inhibition, and induction of CYP1A2, CYP2C8, CYP2C9, CYP2D6, and/or CYP3A4. For these 18 BI proprietary compounds investigated, in vitro CYP inhibition, inactivation, and induction studies were performed in similar fashion as previously reported (Sabo et al. (2015) [10] or Sane et al. (2016) [11]). A brief outline of the methodology is described below.

### 2.1. CYP Inhibition Studies

The IC_50_ values for BI developmental compounds were determined using standard inhibition assays as previously described [10,11,12,13,14]. In brief, test articles were incubated at 37 °C with relevant substrate (i.e., midazolam, dextromethorphan, diclofenac, S-mephenytoin), and human liver microsomes (HLMs).

### 2.2. CYP Induction Studies

CYP3A induction parameters were determined using sandwich-cultured primary human hepatocyte as described [11,14]. In brief, hepatocytes were treated with test articles for 48 h. Induction was assessed via two methods: quantification of 6β-hydroxytestosterone and mRNA level. Test articles were compared against the control (25 μM rifampin).

The IC_50_ values were generated using CYP isoform-specific reactions in HLMs. The following CYP probe substrates were used at concentrations equal to the apparent Michaelis–Menten kinetic constant (K_m_) of that substrate: phenacetin (CYP1A2), bupropion (CYP2B6), diclofenac (CYP2C9), (S)-mephenytoin (CYP2C19), bufuralol (CYP2D6), and midazolam/testosterone (CYP3A). Each isoform-specific CYP probe substrate was incubated in the presence of various concentrations of the BI test compounds with pooled HLM in potassium phosphate buffer and respective isoform-specific metabolites, and respective isoform-specific metabolites were monitored.

### 2.3. CYP Inactivation Studies

The methods used were published previously [10,11,13]. In brief: The BI compounds were assessed for their potential to inactivate CYP2B6, CYP2C9, CYP2D6, and CYP3A using recombinant human CYP isoforms (rCYP) or HLMs. The following CYP probe substrates were used: bupropion (CYP2B6), diclofenac (CYP2C9), dextromethorphan (CYP2D6), and midazolam/testosterone (CYP3A). Primary incubations consisted of buffer, rCYP or HLMs, and various concentrations of the 18 BI test compounds. Samples were preincubated at 37 °C for 5 min. Reactions were initiated by adding NADPH (2 mM), with control reactions conducted without NADPH. At specified time points (0–35 min), a 50 µL aliquot was removed and diluted 1:20 into secondary incubation wells, which contained the isoform-selective probe substrate. After the secondary incubation (37 °C, 6 min), samples were filtered using centrifugation and filtrates were analyzed using liquid chromatography–tandem mass spectrometry. Rate constants for loss of CYP activity, normalized to the corresponding controls without the test compound, were used in Equation (1) to obtain inactivation parameters:

(1) $$k_{obs}=\frac{k_{inact}\times [I]}{K_{I}+[I]}$$

where k_obs_ is the observed rate constant for inactivation, k_inact_ is the maximal inactivation rate constant, [I] is the concentration of inactivator in the primary incubation, and K_I_ is the concentration of inactivator at which the rate of inactivation is half maximal.

### 2.4. P450 Induction and Cytotoxicity

The methods used were published previously [10,11,14]. In brief: To assess induction of CYP1A2, CYP2B6, and CYP3A4 mRNA, three separate lots of cryoplateable human hepatocytes, precharacterized for prototypical induction response, were used. When concentration dependent induction was observed, the data were fitted to determine EC_50_ values using Equation (2):

(2) $$E_{max}=Bottom+\frac{\left(Top-Bottom\right)}{1+10^{\left(({LogEC}_{50}-X)\times Hillslope\right)}}$$

where X is the logarithm of concentration, Top is the maximal response, and Bottom is the baseline value.

### 2.5. Clinical Data

Clinical DDI data (AUC, C_max_) were obtained from BI clinical trial reports.

Informed consent was obtained from all participants of the clinical trials and, and all clinical studies were conducted in accordance with the Declaration of Helsinki and approved by the respective Institutional Review Board.

Various static and mechanistic models suggested by the FDA guidance [5] were applied to retrospectively evaluate the DDI potential.

### 2.6. Basic Static Models

The basic static models for reversible inhibition were used according to the FDA guidelines [5] to determine if the compound is an inhibitor of metabolizing enzymes.

In short, for reversible inhibition, the ratio (R_1_) of intrinsic clearance values for an enzymatic pathway in the absence and in the presence of the interacting drug was calculated as follows:

$$R_{1}=1+\frac{I_{max,u}}{K_{i,u}}$$

where I_max,u_ is the maximal unbound plasma concentration of the interacting drug at steady state and K_i,u_ is the unbound inhibition constant determined in vitro.

For time-dependent inhibition, the ratio R_2_ was calculated as

$$R_{2}=\frac{{(k}_{obs}+k_{deg})}{k_{deg}}$$

where $k_{obs}=\frac{k_{inact}\times 50\times I_{max,u}}{K_{I,u}\times 50\times I_{max,u}}$; k_obs_ is the observed (apparent first order) inactivation rate of the affected enzyme; k_deg_ is the apparent first-order degradation rate constant of the affected enzyme; k_inact_ is the maximal inactivation rate constant; K_I,u_ is the unbound inhibitor concentration causing half-maximal inactivation; and I_max,u_ is the maximal unbound plasma concentration of the interacting drug at steady state.

A compound is regarded to have a DDI potential if R_1_ ≥ 1.02 or R_2_ ≥ 1.25.

For determination, if the compound is an inducer of metabolizing enzymes, the following ratio of intrinsic clearance values of a probe substrate for an enzymatic pathway in the absence and presence of an inducer (R_3_) was calculated:

R3=11+d×Emax×10×Imax,uEC50+10×Imax,u

where d is the scaling factor and is assumed to be 1, E_max_ is the maximum induction effect determined in vitro, I_max,u_ is the maximal unbound plasma concentration of the interacting drug at steady state, and EC_50_ is the concentration causing half-maximal effect determined in vitro. A compound is regarded to have a DDI potential if R_3_ ≤ 0.8.

### 2.7. MSM

Static mechanistic models incorporate more detailed drug disposition and drug interaction mechanisms for both interacting and substrate drugs [5].

(3) $$\frac{{AUC}_{i}}{{AUC}_{r}}=\left(\frac{1}{\left[A_{g}\times B_{g}\times C_{g}\right]\times (1-F_{g})+F_{g}}\right)\times \left(\frac{1}{\left[A_{h}\times B_{h}\times C_{h}\right]\times f_{m}+(1-f_{m})}\right)$$

The Equation assumes that the drug has negligible extrahepatic clearance. AUC_i_ is the AUC in presence of the investigational drug; AUC_r_ is the AUC in absence of the investigational drug; A is the effect of reversible inhibitions; B is the effect of time-dependent inhibition; C is the effect of induction; F_g_ is the fraction available after intestinal metabolism; and f_m_ is the fraction of hepatic clearance of the substrate mediated by the CYP enzyme that is subject to inhibition/induction:

$$f_{m}=\frac{\sum_{k=1}^{p}{Cl}_{int,uk(rEi)}\times {SF}_{ki}}{\sum_{k=1}^{p}{Cl}_{int,uk(rEj)}\times {SF}_{kj}}$$

where subscript ‘h’ denotes liver and subscript ‘g’ denotes gut.

Each value can be estimated with the following Equations:

**Gut****Liver**Reversible inhibition$A_{g}=\frac{1}{1+\frac{{\left[I\right]}_{g}}{K_{i}}}$$A_{h}=\frac{1}{1+\frac{{\left[I\right]}_{h}}{K_{i}}}$Time-dependent inhibition$B_{g}=\frac{k_{deg,g}}{k_{deg,g}+\frac{{\left[I\right]}_{g}\times k_{inact}}{{\left[I\right]}_{g}+K_{I}}}$$B_{h}=\frac{k_{deg,h}}{k_{deg,h}+\frac{{\left[I\right]}_{h}\times k_{inact}}{{\left[I\right]}_{h}+K_{I}}}$Induction$C_{g}=1+\frac{d\cdot E_{max}{\cdot \left[I\right]}_{g}}{{\left[I\right]}_{g}+{EC}_{50}}$$C_{h}=1+\frac{d\cdot E_{max}\cdot {\left[I\right]}_{h}}{{\left[I\right]}_{h}+{EC}_{50}}$

where
${\left[I\right]}_{h}=f_{u,p}\times \left(C_{max}+\left(F_{a}\times F_{g}\times k_{a}\times Dose\right)/Q_{h}/R_{B}\right)$;
${\left[I\right]}_{g}=F_{a}\times k_{a}\times Dose/Q_{en}$;
f_u,p_ is the unbound fraction in plasma;
C_max_ is the maximal total (free and bound) inhibitor concentration in the plasma at steady state;
F_a_ is the fraction absorbed after oral administration; a value of 1 should be used when the data are not available;
F_g_ is the fraction available after intestinal metabolism; a value of 1 should be used when the data are not available;
k_a_ is the first order absorption rate constant in vivo; a value of 0.1 min^−1^ can be used when the data are not available;
Q_en_ is the blood flow through enterocytes (e.g., 18 L/h/70 kg);
Q_h_ is the hepatic blood flow (e.g., 97 L/h/70 kg);
R_B_ is the blood-to-plasma concentration ratio;
d is the scaling factor and is assumed to be 1. A different value can be used if supported by prior experience with the system used.

The Drug–drug Interaction Risk Calculator (DDRIC) (PharmaPendium, Elsevier, Amsterdam, The netherlands) [15], developed based on the FDA model, is a computer system used to assess the MSM. This tool can calculate a predicted DDI between proprietary substances and marketed drugs. The DDRIC is built based on published results from in vitro and in vivo experiments in the drug library in the program. If the proprietary drug is a victim (substrate), the DDI calculation is made against perpetrators and related data: (K_i_, IC_50_, k_inact_, EC_50_, E_max_, C_max_, etc.) from the DDIRC database. If the proprietary compound is a perpetrator (inhibitor and/or inducer), the calculation is made with victims and the corresponding mandatory data: fm(E), Fg. The result is a profile of drug–drug interactions between the proprietary drug and potential co-medication drugs on the market.

**Figure 1:**
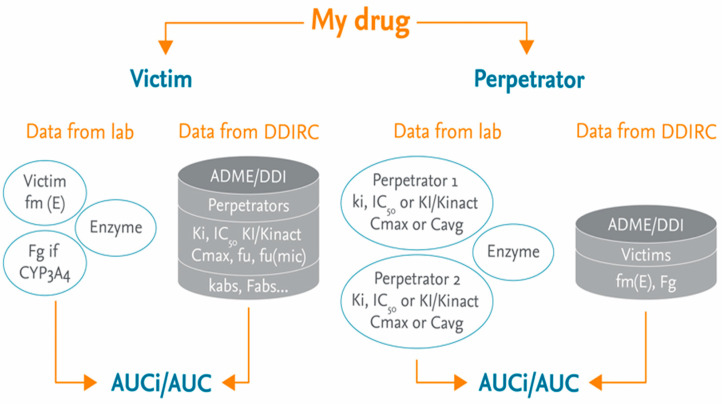
Illustration of the DDIRC workflow for victim and perpetrator drugs (from Ref. [15]).

The DDIRC Drug Library mainly includes drugs that are on the market, but also includes a small number of drugs that are withdrawn. More than 400 perpetrators and 200 victims, along with their own in vitro and in vivo data, are included in the DDIRC. This Drug Library covers the most popular cytochrome P450s (CYP3A4, 2D6, 2C9, 2C19, 2C8, 2B6, 1A2, and 2E1) and, to a lesser extent, UGTs involved in the metabolism of drugs on the market. The Drug Library covers more than 100 therapeutic classes, including the most commonly prescribed ones. The workflow is depicted in Figure 1.

Input parameters for the BI compounds, i.e., inhibition (K_i_, IC_50_, K_I_, K_inact_) and induction (E_max_, EC_50_) parameters, were derived from standard metabolic assays (conducted in HLMs and/or hepatocytes) as described above. Pharmacokinetic parameters (e.g., C_max_, AUC_0–-t_, half-life (t_1/2_)), determined via noncompartmental analysis, were obtained from in-house clinical studies. BI compounds were assessed as both the object drug and precipitant of reversible inhibition, time-dependent inhibition, and induction of CYP1A2, CYP2C8, CYP2C9, CYP2D6, and/or CYP3A4.

Based on the idea from Miano et al. [8], a comparison was made between the predicted DDI potential when this was calculated via the basic MSM method as in the DDIRC and when this MSM was enhanced by incorporating human excretion data or excretion data of preclinical species.

In the latter case, the MSM formula,

$$\frac{{AUC}_{i}}{{AUC}_{r}}=\left(\frac{1}{\left[A_{g}\times B_{g}\times C_{g}\right]\times (1-F_{g})+F_{g}}\right)\times \left(\frac{1}{\left[A_{h}\times B_{h}\times C_{h}\right]\times f_{m}+(1-f_{m})}\right)$$

is changed such that f_m_ (the fraction of hepatic clearance of the substrate mediated by the CYP enzyme that is subject to inhibition/induction) now also incorporates human urine excretion data:

$$f_{m}=\frac{\sum_{k=1}^{p}{Cl}_{int,uk(rEi)}\times {SF}_{ki}}{\sum_{k=1}^{p}{Cl}_{int,uk(rEj)}\times {SF}_{kj}}\times \left(1-f_{e}\right)$$

in which f_e_ is the fraction excreted as parent compound.

Comparison of model predictability was calculated using the following formulas:

- Root Mean Square Error (RMSE) [7]:

$$RMSE=\sqrt{\frac{\sum {\left(predicted\;DDI-observed\;DDI\right)}^{2}}{Number\;of\;predictions}}$$

- Geometric Mean Fold Error (GMFE) [16]:

$$GMFE=10^{\left(\sum_{i=1}^{n}\left|{log}_{10}\left(\frac{{PKparameter}_{predicted,i}}{{PKparameter}_{observed,i}}\right)\right|\right)/n}$$

## 3. Results

In this investigation, a total of 44 clinical studies, encompassing 18 BI proprietary compounds, was used to assess the predictive performance of basic and MSMs outlined in the FDA guidance on in vitro DDI studies [5]. This clinical dataset includes small molecules with diverse range of physicochemical properties and various therapeutic areas (Table 1).

Table 2 and Table 3 summarize the input parameters of these BI development compounds as a perpetrator and a victim of a DDI in various models, respectively.

To verify the predicted DDI potential of the compounds, comparisons between the predicted value and the observed data from clinical studies are necessary. Therefore, an overview of the clinical studies, describing the compound, the interacting drug, the dose and dosing regimen for studies where the BI test compound is a perpetrator or a victim, as well as some characteristic of the population in the study, are depicted in Table 4.

The pharmacokinetic data were obtained in clinical studies in which either single-dose or multiple-dose treatment was given. To quantify the accuracy of our predictions, we compared several parameters between the predicted values and the observed values, including the numbers of true positives, true negatives, false positives, and false negatives, along with specificity, sensitivity, the percentage of predictions within a 2-fold range, RMSE, and GMFE values, as summarized in Table 5.

**Figure 2:**
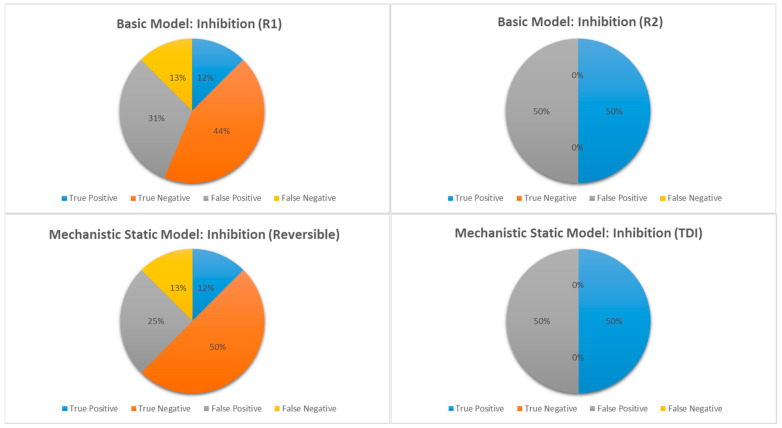
Comparison of true positive, true negative, false positive and false negative DDI prediction for inhibition for BI compounds as perpetrator, when calculated using basic static or mechanistic static models.

**Figure 3:**
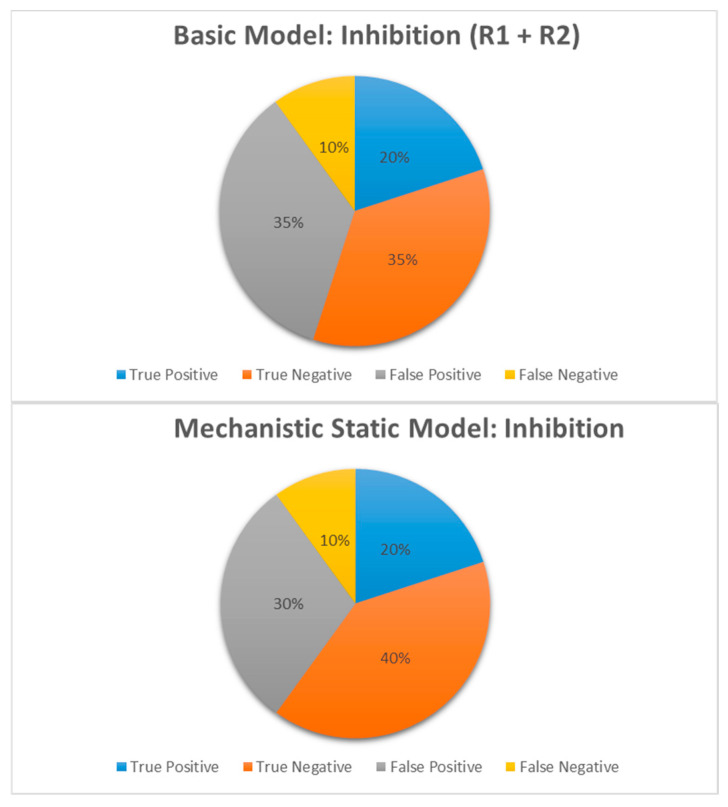
Comparison of true positive, true negative, false positive and false negative DDI prediction for induction for BI compounds as perpetrator, when calculated using basic static or mechanistic static models.

**Figure 4:**
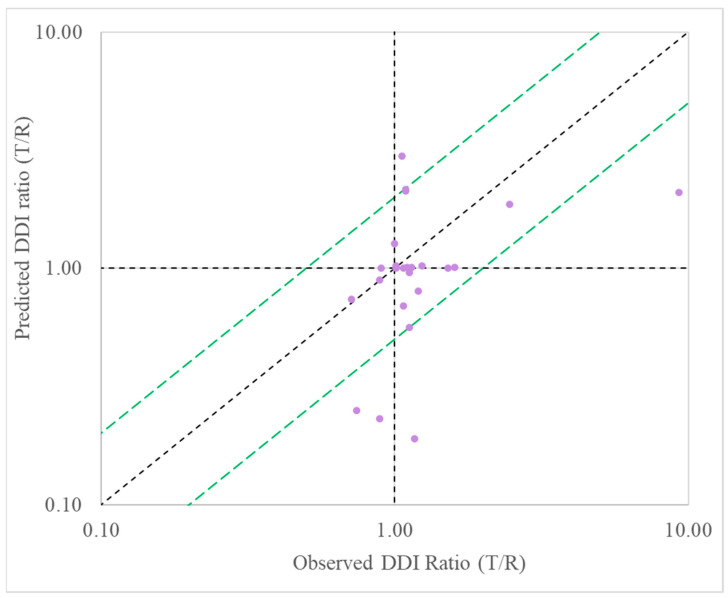
Prediction of DDI for BI developmental compounds as a perpetrator with MSM by comparing predicted and observed DDI ratios. The black dotted line is the line of unity, and the green dotted lines on either side of the line of unity represent a two-fold boundary. The quadrants of the graph depict true or false negatives and positives with respect to inhibition and induction. T = treatment (with interaction drug); R = reference (without interaction drug); DDI ratio was based on AUC.

**Figure 5:**
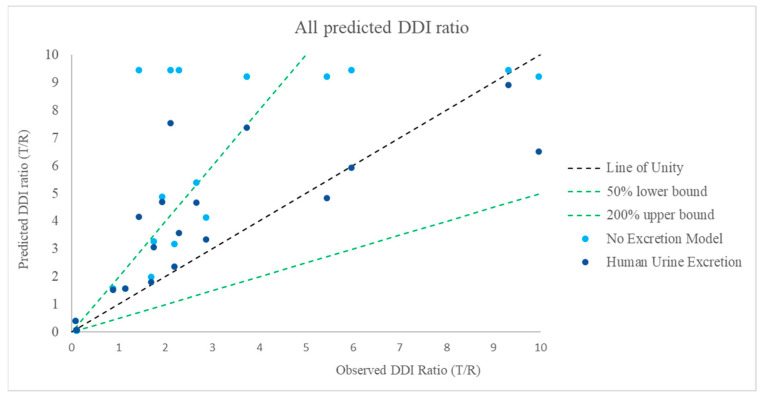
Comparison of predicted and observed DDI ratio for BI development compound as a victim: effect of inclusion of excretion in urine in humans in MSM (The green dotted lines on either side of the line of unity represent a two-fold boundary (the upper green dotted line indicates 200% upper bound; the lower green dotted line indicates 50% lower bound).

**Figure 6:**
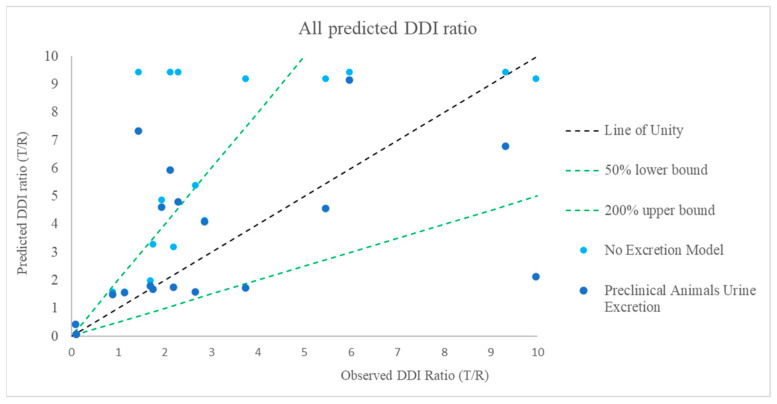
Comparison of predicted and observed DDI ratio for BI development compound as a victim: effect of inclusion of preclinical animal excreta in MSM, (The green dotted lines on either side of the line of unity represent a two-fold boundary (the upper green dotted line indicates 200% upper bound; the lower green dotted line indicates 50% lower bound).

In addition to Table 5, comparisons of the predictive performance of various predictive methods for BI compound as a victim or a perpetrator are depicted in Figure 2, Figure 3, Figure 4, Figure 5 and Figure 6.

Basic vs. mechanistic static model: A comparison of the basic static models, with the calculation of R_1_ and or R_2_, and the mechanistic static models (MSMs), with the BI compound as perpetrator, is graphically shown in Figure 2 and Table 5.

While the false negative rate remained the same at 12% (R_1_), the percentage of false positive predictions was significantly lower, decreasing from 31% to 25% when calculated with the MSM (competitive inhibition). For combined competitive and mechanism-based inhibition (n = 20), the specificity increased from 50% to 57%, while the sensitivity remained at 66.7% (Table 5). Despite the limited sample size (n = 8), the MSM eliminated false negatives for induction (Table 5, Figure 3), though there was an increase in false positive predictions, as shown in Figure 3 and Table 5. For induction, the sensitivity markedly increased from 50% to 100%, while the specificity decreased from 50% to 30% (Table 5). Notably, for induction, two compound–enzyme pairs predicted to be positive in DDIs with the basic model were predicted as negative in DDIs with the MSM. Clinical data confirmed that the induction DDI was negative. These results demonstrated that MSMs should be used for the decision to perform clinical studies to avoid unnecessary DDI studies.

Predictive performance for BI compounds as perpetrator: When evaluating BI compounds as the perpetrator in DDIs, the MSM model successfully predicted the exposure ratio (AUC in the presence-to-absence of inhibitor) to within 2-fold of that observed in clinical studies for 80% for all inhibitors, 50% for inducers, and 75% for all perpetrators (Table 5, Figure 4). This demonstrated a good performance of MSMs in the prediction of DDIs for the prediction of inhibition.

Predictive performance for BI compounds as victim: When calculating the effects with the BI compound as a victim, two methods were used for the MSM: with or without considering the fraction of the compound excreted in human urine, or the corresponding data in preclinical species, such as rats. A clear difference of the predicted values between values from the two methods was observed, as illustrated in Figure 5 (with human excretion vs. without) and Figure 6 (with preclinical animal excretion vs. without). The MSM successfully predicted the exposure ratio (AUC in the presence-to-absence of inhibitor) within 2-fold of that observed in clinical studies for 60% of the compounds (Table 5). The inclusion of the urinary excretion data from Phase I studies (i.e., inclusion of the factor (1 − f_e_) in the formula) resulted in 75% of the tested compounds falling within the 2-fold range (Table 5); if included in the excreta from preclinical species, 60% of the AUC ratios were within a 2-fold range (Table 5). However, based on the criteria of RMSE and GMFE to evaluate the predictive performance, MSM + human urine data was better than MSM + preclinical species excretion, while they were all better than MSM alone (Table 5). Since GMFE < 2 indicates a good predictive performance [16], except for the MSM only model, the other two models with excreta both had GMFE < 2 (Table 5).

## 4. Discussion

The interest in reducing the costs of development of new drugs and reducing the failure rate during the development process have been growing for several decades. One of the factors leading to the early failure of the development of new drugs is the degree of pharmacokinetic DDIs between the developing drug and any comedication that is being taken by the patient.

Many researchers from academia and pharmaceutical industry have been trying to improve pharmacokinetic DDI predictions as early as possible in the development of new drugs. It is commonly believed that IVIVE is the best technique to determine whether a compound has a risk for DDIs. Over the years, several methods and formulas have been used to calculate the risk for DDIs. Extensive investigations on the parameters to include in the formulas for the calculations of DDI predictions have been performed more than 10 years ago by Fahmi et al., who developed a more comprehensive mathematical model that accounts for the simultaneous influences of competitive inhibition, time-dependent inactivation, and induction of CYP3A in both the liver and intestine [17], and a model that simultaneously combines reversible inhibition, time-dependent inactivation, and induction data with static estimates of relevant in vivo concentrations [18], which both served as the input for the rules in the FDA guidelines for DDIs published in 2012 (draft guideline) and 2020 (final guidelines). Subsequently, Yoshida et al. [19] pointed out that for a more accurate and quantitative prediction of DDIs based on in vitro observations, sophisticated model-based predictions, such as PBPK modeling, which incorporate mechanistic considerations of victim and perpetrator drug disposition, should be explored, especially for DDIs involving multiple elimination pathways.

The FDA’s 2012 draft DDI guidance and the subsequent 2020 DDI guidance recommended the use of both basic and mechanistic models (BSMs and MSMs) to predict DDIs; the guidelines were based on studies with approved drugs that were developed decades ago. To our knowledge, our study is the first to systematically evaluate the predictive performance of these FDA-recommended models using proprietary development compounds. This approach provides a much-needed “reality check” on the applicability of these formulas in current drug development settings, highlighting the performance differences between BSMs and MSMs—an analysis that has not been performed before with development-stage compounds. Our findings offer significant insights for industry peers and regulatory bodies, and may inform future refinements to DDI prediction guidance. There are two papers published more than 13 years ago that also used proprietary drugs (Shardlow et al. (2011) [3] and Peterset al. (2011) [4]); however, they were published before the FDA published their 2012 draft guidance; thus, they do not entirely follow the FDA-recommended methods.

By evaluating current FDA DDI formulas with proprietary development compounds, we offer the scientific community valuable data on how these models perform with novel and emerging drug classes. This extension beyond traditionally marketed drugs allows scientists to assess the robustness and applicability of FDA-recommended models across a broader spectrum of chemical entities. Our study could thus catalyze further research and collaborative improvements, refining pharmacokinetic DDI predictions for a wide array of new compounds.

To test for improvement of the prediction via IVIVE, a collection of 18 BI proprietary small molecule compounds which had been developed at least until Phase I clinical studies were selected (Phase I given that human PK data have been accrued for these compounds). Pharmacokinetic parameters (e.g., C_max_, AUC_0–t_, t_1/2_), were determined via noncompartmental analysis in in-house clinical studies. This clinical dataset included small molecules with a diverse range of physicochemical properties and various therapeutic areas. Basic in vitro data, including the IC_50_, K_i_, EC_50_, and E_max_ for specific CYP enzymes, have been collected in earlier studies for these compounds.

The potential for pharmacokinetic DDIs was then calculated according to the FDA guidelines for the BI compounds as a perpetrator or a victim of reversible inhibition, time-dependent inhibition, and induction of CYP1A2, CYP2C8, CYP2C9, CYP2D6, and/or CYP3A4. The guidelines describe basic methods of calculation as well as an MSM. Within our group, we thought that incorporating more extensive data related to the degree of excretion of the compound in humans may influence the prediction accuracy. The DDIRC [15], which was developed based on the FDA MSM, was used to assess the MSM.

For each set of data, we applied both basic static models (BSMs) and mechanistic static models (MSMs) in drug–drug interaction predictions. The basic static models (BSMs R1, R2, and R3) are easy to apply and understand, with fewer parameters and assumptions needed. They calculate risk ratios (R-values) for potential DDIs using readily available data. Due to their simplicity, BSMs save time and resources when flagging possible interaction risks. The BSMs may overestimate or underestimate the DDI risk due to their oversimplified nature. Basic static models do not account for complex interactions, such as multiple enzyme pathways, or specific tissue distributions.

MSMs provide a deeper mechanistic understanding of the DDI, helping to identify which specific enzymes are involved, thereby offering insights into mitigating strategies (e.g., dose adjustment, alternative pathways). MSMs can handle more complex interactions, including competitive and non-competitive inhibition, and multiple metabolic pathways. This flexibility enhances their predictive power for drugs with complex metabolic pathways. MSMs require extensive data on enzyme kinetics, tissue distribution, and sometimes transporter activity, which may not always be readily available in early drug development stages. MSMs are computationally more intensive and may require more time and resources to implement and interpret accurately. With numerous parameters involved, mechanistic models carry a risk of overfitting to specific data sets, which may reduce the generalizability of the predictions to broader populations or different study conditions.

For the prediction of DDIs, from a regulatory agencies point of view, it is important to minimize the false negative prediction, as it leads to missed potential clinical DDIs; on top of that, a reduction in the false positive rate is beneficial as it leads to unnecessary clinical DDIs [20]. In our set of data, despite a small sample size, we have shown that for induction (n = 8), the MSM significantly reduces (eliminates) the false negative rate (sensitivity = 100%) (Figure 3). For inhibition (n = 20), while the false negative rate remains the same, the MSM significantly reduced the false positive rate (Figure 2). These results demonstrated that MSM should be used for the decision to perform clinical studies. These results appear to be consistent with the findings of Einolf [21] using approved drugs.

Although the MSM provided greater prediction accuracy, the input required to generate robust extrapolations can be inefficient and costly. The current study demonstrated that the basic models for drug interactions should be used in early drug discovery to “rule out” DDI risks because of the minimal inputs required and the low rate of false negative predictions. Mechanistic static models can then be implemented for compounds requiring additional evaluation, whereas clinical studies are reserved only for compounds predicted to have DDI liabilities in both models.

For BI compound as the perpetrator, if judged based on GMFE and RMSE values, as compared to the literature [7,12,17] using approved drugs, our prediction based on the FDA formula using BI development compounds had a good prediction performance for all perpetrators, all inhibitors, and all inducers (Table 5). The only GMFE value > 2 was associated with induction (Table 5); for all others, the GMFE values < 2, and GMFE < 2 indicates a good predictive performance [16]. In BI, we took a conservative approach and fixed the d value to 1 for the prediction of induction [7,22], which may lead to a slightly higher GMFE value. In the investigation, the MSMs successfully predicted the exposure ratio (AUC in the presence-to-absence of inhibitor) to within 2-fold of that observed in clinical studies for 80% for all inhibitors, 50% for inducers, and 75% for all perpetrators (Table 5, Figure 4). This also demonstrated the good performance of MSM (based on the FDA formula) using BI development compounds.

In this investigation, we have demonstrated that including excretion data from preclinical species significantly improves the predictive performance for BI compounds (as victim) compared to not using excretion data (Table 5, Figure 6). At the preclinical development stage, only preclinical data, such as the percentage contribution of a specific CYP enzyme to overall metabolism and excretion data (e.g., rat urine and bile excretion), were available for the development compounds. Using preclinical excretion data as a surrogate for human urine data yields a better predictive performance based on GMFE and RMSE values comparable to those obtained with human urine data (Table 5). This method can help the project teams select candidate drugs for clinical development.

Recently, machine learning-based computational models have been applied in the prediction of DDIs (e.g., ref. [23]). Computational models using machine learning analyze large datasets, including pharmacokinetics, pharmacodynamics, biochemical pathways, and molecular interaction data. They can discover hidden patterns and interactions that may not be readily apparent through traditional rule-based methods, which have higher screening efficiency and integrate heterogeneous data sources to build predictive frameworks to predict DDIs. However, these computational models may raise concerns of potentially inconsistent data sources, data imbalance issues, overfitting, high complexity, and resource intensity, etc. Therefore, computational models may play a complementary role, not a replacing role, to traditional working models (BSMs and MSMs) in predicting DDIs by offering enhanced efficiency, accuracy, and insights into complex biological interactions.

It is acknowledged that the calculations performed have some limitations, as the dataset used is somewhat biased for three reasons. First, the compounds that were identified as being most potent inhibitors in in vitro studies did not make it to the clinical stage of development, because for various reasons they had a poor outlook as drug candidates. Secondly, since the “no-effect” range in the FDA guideline is very narrow (0.8–1.25), only a limited amount of data fell within the true negative category. Furthermore, because of a lack of metabolite characterization for the 18 compounds in the study, a metabolite-mediated DDI was not considered as part of the calculations. The small sample size, in particular for induction (n = 8) and mechanism-based inhibition (n = 4), is a limitation of this research.

In summary, the current research evaluated the performance of the FDA static models in predicting pharmacokinetic DDI risks using BI proprietary compounds. The DDIRC (PharmaPendium^®^) was used to assess the MSMs and data were correlated with human pharmacokinetic studies from Phase I clinical trials. The results showed that the FDA formulas for both basic static modes and mechanistic static models performed well in an industrial setting in support of drug development to predict DDIs. The results also demonstrated that basic models for drug interactions should be used in early drug discovery to “rule out” DDI risks because of the minimal inputs required and the low rate of false negative predictions. The MSMs showed good performance in predicting clinical DDIs and should be implemented for compounds needing further clinical evaluation. Furthermore, the inclusion of human urine or preclinical species excretion data in the formula for the MSM enhanced the predictivity for the DDI potential of (candidate) drugs.

## 5. Conclusions

Our present investigation demonstrated that FDA formulas for both basic static models and mechanistic static models performed reasonably well in an industrial setting in support of drug development to predict pharmacokinetic DDIs. The basic model should be used in the early phases of drug development to rule out the need for a clinical DDI study, as the rate of false negative predictions is negligible. The MSM should be reserved for when further assessments are deemed necessary, as it requires more resources to execute robustly. Furthermore, the inclusion of human urine excretion data in the MSM equation, via integration of factor (1 − *f_e_*), enhanced the predictivity of the DDI potential of (candidate) drugs.

## Figures and Tables

**Table 1:** Physicochemical characteristics of BI compounds in DDI prediction analyses.

Compound Name	Molecular Weight (g/mol)	LogP	pKa	Predominant Ionized Form at pH7.4	Nominal BCS Class	Protein Binding (%)
BI-1	449	2.6	4.8	Basic	IV	76.7%
BI-2	537	3.2	8.6	Neutral	II or IV	96.4%
BI-3	486	3.1	5.1, 6.1	Basic	IV	93.2%
BI-4	525	1.3	4.1, 8.0	Basic	III	86%
BI-5	531	3.5	6.1, 8.5	dibasic	I	85.80%
BI-6	519	2.3	No ionizable center	No ionizable center	II	59.6%
BI-7	525	5.2	No pKa	Neutral Molecule	II	99.75%
BI-8	512	2.1	3.0, 7.4	Basic	III	70%
BI-9	379	2.4	4.4	Neutral	II	89.40%
BI-10	311	1.7	4.2, 9.0	NA	I	27%
BI-11	512	1.2	No pKa	NA	II	80.40%
BI-12	784	2.6	6.5, 10.4	NA	IV	90%
BI-13	353	1.99	3.9, 8.7	NA	I	64.40%
BI-14	526	2.9	2.6 (D360), >11	Basic	II	88.80%
BI-15	521	4.2	4.9, 8.8	Basic	I	88.60%
BI-16	533	3.4	2.2	Basic	II	84.60%
BI-17	382	1.3	<2	NA	I	60.50%
BI-18	606	4.3	8.2	Base	NA	99.88%

NA: not available.

**Table 2:** Parameters of BI compounds as perpetrators in drug–drug interactions.

Compound Name	Enzyme	Competitive Inhibition (µM)	Inactivation		Induction		Dosage	Dose (mg)	C_max_/C_avg_ (ng/mL)	fu
			KI (µM)	Kinact (min)	EC_50_ (µM)	Emax. (Fold)				
BI-6	CYP3A4				17.6	10	Repeated	120	187.88	0.404
BI-7	CYP3A4				0.15	6.1	Single	200	332.79	0.003
	CYP3A4				0.15	6.1	Repeated	200	603.64	
BI-9	CYP1A2	142					Repeated	240	5682.75	0.106
	CYP2C19	16.4								
	CYP2C9	22.3								
	CYP2D6	142								
	CYP3A4	47.3	115	0.0103	1.17	6.45				
BI-11	CYP2C	55 (IC_50_)					Repeated	25	223.93	0.196
	CYP3A4		128	0.0234	1.96	18.7				
BI-12	CYP3A4	10.4 (IC_50_, midazolam)	4.28	0.23			Single	100	349.61	0.100
							Single	500	2539.77	
BI-14	CYP3A4		28.8	0.14	2.09	2.33	Repeated	80	27.64	0.112
BI-15	CYP3A4				0.0231	2.78	Repeated	10	20.79	0.114
BI-16	CYP1A2				3.12	2.35	Repeated	300	1688.34	0.154
	CYP2C9	16.9								
	CYP2C19	50								
	CYP3A4		3.21	0.013	9.76	13.3	Repeated	400	1203.68	
BI-18	CYP1A2	7.95			NA	NA	Single	500	1700	0.01
	CYP2C19	1.24								
	CYP2C9	2.17			NA	NA				
	CYP2D6	4.46								
	CYP3A	0.44			NA	NA				

NA: not available.

**Table 3:** Parameters of BI compounds as victims in drug–drug interactions.

Compound Name	Enzyme	Kmet Without Excretion Correction	Human Excretion	Preclinical Excretion
BI-1	CYP3A	90.00%	19.50%	11.46%
BI-2	CYP3A	70.00%	3.00%	42.09%
BI-3	CYP3A	90.00%	3.00%	52.68%
BI-4	CYP3A	90.00%	5.00%	40.43%
BI-5	CYP3A	90.00%	3.00%	7.00%
BI-6	CYP3A	50.00%	10.00%	11.25%
BI-7	CYP3A	80.00%	1.00%	1.50%
BI-8	CYP3A	90.00%	11.00%	12.35%
BI-10	CYP3A	9.80%	1.00%	2.10%
	CYP2C19	90.20%	1.00%	2.10%
BI-11	CYP3A	90.00%	7.00%	0.36%
BI-13	CYP3A	36.70%	5.00%	10.74%
BI-15	CYP3A	69.00%	16.00%	37.26%
BI-16	CYP3A	90.00%	0.70%	4.60%
BI-17	CYP3A	90.00%	15.00%	3.40%

Note: Kmet = the fraction of the total metabolic clearance mediated by the selected enzyme for the victim drug.

**Table 4:** Overview of the clinical trial design of the drug–drug interaction studies.

**Perpetrators of P450 Inhibition**	**Interacting Drug**	**Perpetrator Oral Dose (mg)**	**Perpetrator Dosing Regimen**	**Victim Oral Dose (mg)**	**Victim Dosing Regimen**	**Fed/Fasted**	**N**	**Age Range (Year)**	**Observed AUCR**
BI-16	midazolam	400	28 days, qd	75 µg	sd, day 14	Fed	9	21–43	1.06
	midazolam	300	20 days, qd	2	sd, day 15	Fasted	16	24–51	1.27
	warfarin	300	20 days, bid	10	sd, day 15	Fasted	16	24–51	1.10
	omeprazole	300	20 days, bid	20	sd, day 15	Fasted	16	24–51	1.00
	caffeine	300	20 days, bid	100	sd, day 15	Fasted	16	24–51	1.12
BI-7	midazolam	200	1 day, qd	75 µg	sd, day 1	Fed	8	18–33	0.90
	midazolam	200	14 days, qd	75 µg	sd, day 14	Fed	8	18–33	0.89
BI-11	midazolam	25	14 days, qd	2	sd, day 10	Fasted	13	23–54	0.71
	warfarin	25	14 days, qd	10	sd, day 10	Fasted	13	23–54	1.01
	omeprazole	25	14 days, qd	20	sd, day 10	Fasted	13	23–54	0.89
BI-14	midazolam	80	17 days, qd	75 μg	sd, day 17	Fed	8	26–51	1.09
BI-6	midazolam	150	11 days, bid	75 μg	sd, day 11	Fed	9	22–65	1.17
BI-15	midazolam	10	14 days, qd	75 µg	sd, day 14	Fasted	8	34–54	1.20
BI-9	midazolam	240	12 days, qd	2	sd, day 7	Fasted	24	18–51	0.74
	warfarin	240	12 days, qd	10	sd, day 7	Fasted	24	18–51	1.01
	omeprazole	240	12 days, qd	20	sd, day 7	Fasted	24	18–51	1.12
	caffeine	240	12 days, qd	100	sd, day 7	Fasted	24	18–51	1.07
	metoprolol	240	12 days, qd	50	sd, day 7	Fasted	24	18–51	1.07
BI-12	midazolam	100	1 day, qd	2	sd, day 1	Fasted	12	21–50	2.46
	midazolam	500	1 day, qd	2	sd, day 1	Fasted	12	21–50	9.28
BI-18	midazolam	500	1 day, qd	0.025 mg/kg	sd, day 1	Fasted	20	23–55	1.09
	warfarin	500	1 day, qd	10	sd, day 1	Fasted	20	23–55	1.52
	caffeine	500	1 day, qd	200	sd, day 1	Fasted	20	23–55	1.14
	omeprazole	500	1 day, qd	40	sd, day 1	Fasted	20	23–55	1.24
	dextromethorphan	500	1 day, qd	30	sd, day 1	Fasted	20	23–55	1.60
**Victims of P450 inhibition**	**Interacting Drug**	**Perpetrator Oral Dose (mg)**	**Perpetrator Dosing Regimen**	**Victim Oral Dose (mg)**	**Victim Dosing Regimen**	**Fed/Fasted**	**n**	**Age Range (year)**	**Observed AUCR**
BI-1	itraconazole	200	12 days, qd	6	sd, day 4	Fasted	16	22–56	2.29
BI-2	itraconazole	200	1 day bid, 6 days qd	25	sd, day 4	Fasted	16	25–47	1.75
BI-3	ketoconazole	200	4 days, bid	25	sd, day 3	Fasted	18	23–50	3.73
	voriconazole	200/400	day 1 400 mg bid, 3 days 200 mg bid	25	sd, day 3	Fasted	18	23–50	2.66
BI-4	ketoconazole	200	5 days, bid	50	sd, day 3	Fasted	16	23–50	9.97
BI-5	itraconazole	200	12 days, qd	2.5	sd, day 4	Fasted	14	22–50	2.11
BI-6	itraconazole	200	10 days, qd	10	sd, day 4	Fasted	14	27–49	1.69
BI-7	itraconazole	200	14 days, qd	10	sd, day 4	Fasted	16	25–45	1.93
BI-8	ketoconazole	200	4 days, bid	200	sd, day 3	Fed	24	21–50	5.45
BI-10	itraconazole	200	1 day bid, 4 days qd	25	sd, day 3	Fasted	24	20–45	1.14
	fluconazole	100/50	1 day 50 mg bid, 2 days 100 mg bid, 1 day 100 qd	10	sd, day 4	Fasted	18	24–50	31.03
	rifampicin	600	8 days, qd	50	sd, day 8	Fasted	15	25–52	0.08
BI-11	itraconazole	200	1 day bid, 9 days qd	25	sd, day 4	Fed	16	24–51	5.97
	rifampicin	600	10 days, qd	25	sd, day 7	Fasted	16	24–55	0.10
	fluconazole	400	13 days, qd	10	sd, day 5	Fasted	15	25–50	2.86
BI-13	itraconazole	200	1 day bid, 9 days qd	10	sd, day 4	Fed	10	26–49	0.88
BI-15	itraconazole	200	14 days, qd	2	sd, day 4	Fasted	14	23–48	2.19
BI-16	itraconazole	200	12 days, qd	50	sd, day 4	Fasted	14	21–51	9.31
BI-17	itraconazole	200	5 days, qd	3	sd, day 4	Fasted	14	25–50	1.43
	rifampicin	600	7 days, qd	50	sd, day 8	Fasted	16	18–50	0.11

**Table 5:** Summary of the predictive performance of basic and mechanistic static model for prediction of drug–drug interaction.

		# of BI Compound-Enzyme Pairs	TP ^a^	TN ^b^	FP ^c^	FN ^d^	Sensitivity ^e^	Specificity ^f^	% of Predictions Within 2-Folds	RMSE ^g^	GMFE ^h^
BI Compound as Perpetrator											
	Basic Static Model (R_1_)	16	2	7	5	2	50.0	58.3	n/a	n/a	n/a
	Basic Static Model (R_2_)	4	2	0	2	0	100.0	0.0	n/a	n/a	n/a
	Basic Static Model (R^1^ + R_2_)	20	4	7	7	2	66.7	50.0	n/a	n/a	n/a
	MSM (for all inhibitions)	20	4	8	6	2	66.7	57.1	80	1.72	1.5
	Basic Static Model (R_3_)	8	1	3	3	1	50.0	50.0	n/a	n/a	n/a
	MSM (only compounds with induction)	8	2	2	4	0	100.0	33.3	50	0.91	2.27
	MSM (all perpetrators)	24	4	10	8	2	66.7	55.6	75	1.59	1.64
BI Compound as Victim											
	MSM	20	18	0	2	0	100.0	0.0	60	6.43	2.12
	MSM + Human Urine	20	18	0	2	0	100.0	0.0	75	5.82	1.74
	MSM + Rat Excretion	20	18	0	2	0	100.0	0.0	60	6.20	1.97

^a^: TP = true positive; ^b^: TN: true negative; ^c^: FP = false positive; ^d^: FN = false negative; ^e^: Sensitivity = percentage actual positivity DDIs predicted as positive = TP/(TP + FN); ^f^: Specificity = percentage actually negative DDIs predicted as negative = TN/(TN + FP); ^g^: RMSE = root mean square error; ^h^: GMFE = geometric mean fold error.

## Data Availability

Data generated or analyzed during this study are included in this published article, further inquiries can be directed to the corresponding author.
